# Chemical characterisation of the vapour emitted by an e-cigarette using a ceramic wick-based technology

**DOI:** 10.1038/s41598-022-19761-w

**Published:** 2022-10-03

**Authors:** M. Isabel Pinto, J. Thissen, N. Hermes, A. Cunningham, H. Digard, J. Murphy

**Affiliations:** 1 B.A.T (Investments) Limited, R&D, Regents Park Rd, Southampton, SO15 8TL UK; 2 Reynolds American, Inc., 401 N Main St, Winston-Salem, NC 27101 USA

**Keywords:** Chemistry, Analytical chemistry

## Abstract

Fourth-generation ‘pod’ e-cigarette devices have been driven by technological advances in electronic atomization of the e-liquid. Use of microporous ceramic as a wicking material improves heating efficiency, but how it affects the chemical emissions of these devices is unclear. We assessed the emissions of a pod e-cigarette with innovative ceramic wick-based technology and two flavoured e-liquids containing nicotine lactate and nicotine benzoate (57 and 18 mg mL^−1^ nicotine, respectively). Among the studied harmful and potentially harmful constituents (HPHCs) listed by the US FDA and/or WHO TobReg, only 5 (acetone, acetaldehyde, formaldehyde, naphthalene and nornicotine) were quantified at levels of 0.14 to 100 ng puff^−1^. In the combustible cigarette (Kentucky reference 1R6F), levels were from 0.131 to 168 µg puff^−1^. Nicotine levels ranged 0.10–0.32 mg puff^−1^ across the 3 study products. From the 19 proposed HPHCs specifically of concern in e-cigarettes, only 3 (glycerol, isoamyl acetate and propylene glycol) were quantified. The low/undetectable levels of HPHCs reflect not only the optimal operating conditions of the e-cigarette, including an efficient supply of e-liquid by the ceramic wick without overheating, but also the potential of the e-cigarettes to be used as an alternative to combustible cigarettes.

## Introduction

E-cigarettes are battery-powered devices designed to deliver nicotine and/or other substances including, in some cases, flavourings. Although e-cigarettes were first proposed in 1927 by Joseph Robinson^1^, it was only in the early 2000s that the 1st generation of e-cigarettes or ‘cig-a-likes’ became commercially available^2–4^. Subsequent generations of devices have evolved since then, ranging from e-cigarettes with prefilled or refillable cartridges (2nd generation) to rechargeable tank-style devices (3rd generation) with modifiable or ‘‘Mods’’ components^3–6^. The 4th generation of devices, known as ‘Pods’, has been driven by advances in electronic atomization technology^3,7–9^.

E-cigarettes consist of a mouthpiece, an e-liquid chamber, an atomiser and a battery. The atomiser has a wicking material that draws the e-liquid onto a battery-powered heating coil. Optimal vapour production depends on an efficient supply of e-liquid to the heating coil, which is limited by the wicking and rate of e-liquid evaporation^10–12^. Power levels that produce aerosol beyond the ability of the wick to resupply the liquid to the coil may result in overheating of the atomizer coil and consequently overheating of the e-liquid^10,11^. Different types of wicking material, varying in size and shape, have been used in e-cigarettes^3,13^. Silica was commonly the first material to be used as a wick, followed by cotton and ceramic^3,13–15^. Cotton has good wicking properties but is less thermally stable than silica^14,16,17^, while ceramic is chemically stable and heat-resistant^18^. The use of microporous ceramic as a wicking material has increased in the past few years^14,16,18–20^. Its application has been reported to improve heating efficiency and reduce charring^14,16,18–20^.

E-liquids are an important part of any vaping system and their composition, together with the characteristics of the device, may have an impact on nicotine delivery^21^. They mainly constitute a mix of propylene glycol (PG), glycerol (vegetable glycerine or VG) and nicotine. E-liquids may include flavouring compounds and usually come in different nicotine strengths or concentrations.

To help adult users to completely switch to alternative nicotine products, it is important the other alternatives provide effective nicotine delivery comparable or close to that of conventional/combustibles cigarettes^22,23^. Heavy smokers (12.4 ± 8.4 cigarettes per day, n = 11) have found that e-cigarettes, especially those from the 1st generation, were unsatisfactory because delivery of nicotine was ineffective as compared with conventional cigarettes^22^. Later generations of devices have achieved improved nicotine delivery by using different product designs and power settings, innovative materials, and nicotine salts in e-cigarette formulations^3,21,22,24,25^. For example, Bowen and Xing^24^ reported that a combination of nicotine with some weak organic acids, such as benzoic, lauric, levulinic, salicylic or sorbic acid, provides satisfaction comparable to that of conventional cigarettes. They suggested that the satisfaction effect was consistent with an efficient transfer of nicotine to the user’s lungs and a rapid rise in nicotine absorption in the plasma^24^. Use of lactic acid and pyruvic acid has been investigated by other authors, who reported nicotine absorption kinetics that are similar to those of conventional cigarettes and associated with acceptable sensory qualities and relief of craving^23,25–27^. A combination of nicotine with weak organic acids to form nicotine salts has also been applied in pharmaceutical formulations used in Metered Dose Inhalers (MDIs) therapy equipment^28^. Its application in e-cigarette formulations has the potential to mimic cigarette smoking’s nicotine pharmacokinetics, which may help cigarette smokers to transition to e-cigarettes^22,23,25–27,29–32^.

E-cigarettes do not burn tobacco and may produce less harmful and potentially harmful constituents (HPHCs) as compared with combustible cigarettes^6,33–37^. HPHCs have been defined by the US Food and Drug Administration (U.S FDA) as chemicals or chemical compounds in tobacco products or tobacco smoke that cause or might cause harm to smokers or non-smokers^38,39^. E-cigarettes have been recognised as an alternative for adult smokers who are unable or unwilling to quit smoking^35,37,40–46^. The most recent Public Health England evidence review highlights, as a key finding, a study suggesting that the cancer potencies of e-cigarettes were largely less than 0.5% of those of smoking^42^. The risks of cardiovascular disease and lung disease have not been quantified for e-cigarettes, but are also likely to be substantially less than those from smoking^42^. Because e-cigarettes do not burn tobacco, the reduction of harmful substances depends on the chemical composition of the e-liquid, as well as the characteristics of the device^4,5,15,47–49^. For example: overheating of e-liquid on the coil and poor wicking performance may lead to an increase in carbonyls to levels higher than observed in cigarette smoke^11,15,47,50,51^.

Compared with silica and cotton wicking materials, there are fewer studies on ceramic wick-based e-cigarette systems, and their impact on e-cigarettes emissions is less documented in the literature. To address this gap, the aim of this study was to characterise the vapour emitted by a 4th-generation pod e-cigarette designed with a ceramic wick-based technology using ISO 20768:2018 standard puffing regime (55 mL puff volume/3 s puff duration/30 s puff frequency; rectangular puff profile)^52^. The emissions of two Berry Blast flavoured e-liquids with different levels of nicotine and different nicotine salts (BB57 with 57 mg mL^–1^ of nicotine containing lactic acid and BB18 with 18 mg mL^–1^ of nicotine containing benzoic acid) were tested for a total of 89 organic compounds covering different classes of compounds (e.g., nicotine and non-nicotine toxicants). From those, 55 compounds have been listed by the U.S. FDA as relevant to tobacco products and with 19 compounds proposed by the FDA as HPHCs of specific concern in e-cigarette aerosols^38,39,44,53,54^. We also focused on the nine toxicants (acetaldehyde, acrolein, benzo[a]pyrene, benzene, 1,3-butadiene, carbon monoxide (CO), formaldehyde, nitrosonornicotine (NNN) and 4-(*N*-nitrosomethylamino)-1-(3-pyridyl)-1-butanone (NNK) recommended for mandated reduction in cigarette smoke by the WHO Tobacco Product Regulation Group (WHO TobReg) which are also part of the HPHCs U.S.FDA list^53–55^. To provide context, e-cigarette vapour emissions were compared with smoke yields from a reference cigarette (Kentucky 1R6F (Ky1R6F)) smoked under ISO 20778:2018 puffing regime (55 mL puff volume/2 s puff duration/30 s puff frequency; bell-shaped puff profile, 100% ventilation blocked)^56,57^.

## Results and discussion

### Carbon monoxide, aerosol mass and water

Table 1 summarizes the per-puff levels of CO, aerosol collected mass (ACM), water and nicotine in the emissions from two e-cigarettes: namely, Berry Blast 57 mg mL^−1^ of nicotine containing lactic acid (BB57); and Berry Blast 18 mg mL^−1^ of nicotine containing benzoic acid (BB18). CO, which is associated with combustion of organic material, was below the limit of detection (< LOD) for both e-cigarettes, with a percentage reduction of 99.8% relative to Ky1R6F cigarette smoke (Table 1). ACM, which comprises mainly PG, VG, water, nicotine and other minor constituents, was in the same range for both e-cigarettes. ACM results were found to be reproducible across all methods as demonstrated by the low standard deviation of ACM in both e-cigarette emissions (6.58 ± 0.39 mg puff^−1^ and 6.46 ± 0.36 mg puff^−1^ for BB57 and BB18 respectively), accounting for a coefficient of variation of 5.9% and 5.5% for BB57 and BB18, respectively (n = 85). This is an indication of sampling robustness and puffing consistency. The nicotine-free dry particulate matter (NFDPM) or ‘tar’, a parameter associated with cigarette smoke, consists predominantly of combustion by-products^36,58^. The level of NFDPM, 3.67 ± 0.30 mg puff^−1^ equivalent to 33 ± 3 mg cig^−1^, was in accordance with the Ky1R6F certified value of 29 ± 2 mg cig^−1^ (ISO Intense smoking regime)^56^.

**Table 1:** CO, ACM, NFDPM, water, nicotine, humectants and related impurities and toxicants: per-puff concentration of e-cigarette vapour emissions, respective method air blanks and Ky1R6F cigarette smoke.

Vapour constituent	Unit	ePod1.0			Cigarette	Percentage reduction (%)	
		Air blank	BB57	BB18	Ky1R6F	BB57	BB18
**CO, ACM, NFDPM, water and nicotine**							
Puff count	Per consumable	50	50	50	9	-	-
CO	mg puff^–1^	< LOD	< LOD	< LOD	3.02 ± 0.13	99.8	99.8
ACM	mg puff^–1^	-	6.58 ± 0.39	6.46 ± 0.36	-	-	-
NFDPM	mg puff^–1^	-	-	-	3.67 ± 0.30	-	-
Water	mg puff^–1^	< LOD	0.46 ± 0.01	0.44 ± 0.02	2.12 ± 0.19	-	-
Nicotine	mg puff^–1^	< LOD	0.32 ± 0.01	0.10 ± 0.01	0.23 ± 0.01	-	-
**Humectants**							
Puff count	Per consumable	50	50	50	9	-	-
Propylene glycol	mg puff^–1^	0.01 ± 0.01	2.31 ± 0.10	2.60 ± 0.19	0.04 ± 0.002	-	-
Glycerol	mg puff^–1^	< LOD	3.27 ± 0.13	3.46 ± 0.24	0.19 ± 0.01	-	-
Diethylene glycol	mg puff^–1^	< LOD	< LOD	< LOD	< LOD	NC	NC
Ethylene glycol	mg puff^–1^	< LOD	< LOD	< LOD	0.01 ± 0.003	99.6	99.6
Glycidol	mg puff^–1^	< LOD	< LOD	< LOD	< LOD	NC	NC
**Nicotine-related impurities**							
Puff count	Per consumable	50	50	50	9	-	-
Anabasine	µg puff^–1^	< LOD	< LOD	< LOD	< LOQ	NC	NC
Anatabine	µg puff^–1^	< LOD	< LOD	< LOD	0.13 ± 0.004	98.2	98.2
Cotinine	µg puff^–1^	< LOD	< LOQ	0.01 ± 0.001	0.32 ± 0.01	98.3	97.7
Myosmine	µg puff^–1^	< LOD	< LOQ	0.03 ± 0.02	0.23 ± 0.02	91.5	85.0
Nicotine-*N*-oxide	µg puff^–1^	< LOD	< LOQ	< LOD	< LOD	NC	NC
β-Nicotyrine	µg puff^–1^	< LOD	< LOQ	< LOQ	0.42 ± 0.01	98.0	97.8
Nornicotine	µg puff^–1^	< LOD	0.05 ± 0.003	0.03 ± 0.001	0.28 ± 0.03	81.6	90.7
**Tobacco-specific nitrosamines**							
Puff count	per consumable	50	50	50	9	-	-
Nitrosonornicotine (NNN)	ng puff^–1^	< LOD	< LOD	< LOD	25.8 ± 0.90	> 99.9	> 99.9
Nitrosoanabasine (NAB)	ng puff^–1^	< LOD	< LOD	< LOD	2.43 ± 0.13	99.9	99.9
Nitrosoanatabine (NAT)	ng puff^–1^	< LOD	< LOD	< LOD	30.5 ± 1.39	> 99.9	> 99.9
4-(*N*-nitrosomethylamino)-1-(3-pyridyl)-1-butanone (NNK)	ng puff^–1^	< LOD	< LOD	< LOD	20.9 ± 0.70	> 99.9	> 99.9

Values are presented as mean ± standard deviation (n = 5) with the exception of ACM (n = 85). Percentage reduction of e-cigarette emissions relative to Ky1R6F cigarette smoke. *CO* carbon monoxide, *ACM* aerosol collected mass, *NFDPM* nicotine-free dry particulate matter, *NC* not calculated, *LOD* limit of detection, *LOQ* limit of quantification. More information about the analytical methods and respective LODs and LOQs are under the Supplementary Information (Tables S1 and S2).

### Humectants and related impurities

In terms of humectants, levels of PG and VG were higher in the e-cigarette emissions than in the Ky1R6F cigarette smoke (Table 1). Because PG and VG are the main constituents of e-liquids, these results were expected. Diethylene glycol (DEG) and ethylene glycol (EG), which may be present in e-liquids as impurities^59,60^, were < LOD in the e-cigarette emissions. These compounds were raised as a potential concern by the U.S FDA after reports of their detection in e-liquids^53,54,61^. EG is widely used as an anti-freeze agent and is associated with pronounced toxicological risks^50^. The US Pharmacopeia (USP) has set a limit for DEG and EG of 0.1% (1 mg g^–1^) in both PG and VG^59,60,62^. Their low levels in the e-cigarette emissions shows the importance of using pharmaceutical-grade PG and VG. Glycidol, which is listed as a probable carcinogen by the International Agency for Research on Cancer (IARC)^63^, was < LOD for both e-cigarette emissions and cigarette smoke.

### Nicotine, nicotine-related impurities and TSNAs

As shown in Table 1, different nicotine concentrations were observed for the e-cigarette emissions and Ky1R6F cigarette smoke, with nicotine levels in cigarette smoke (0.23 mg puff^–1^) lying between those in the two e-cigarette emissions (BB18, 0.10 mg puff^–1^; BB57, 0.32 mg puff^–1^). The concentration of nicotine in the BB57 emissions relative to BB18 was three times greater and followed the three-fold increase in nicotine strength of the e-liquid. In the cigarette smoke, the measured nicotine concentration of 0.23 ± 0.01 mg puff^–1^ (Table 1), equivalent to 2.07 ± 0.09 mg cig^−1^, is in accordance with the Ky1R6F certificate value of 1.9 ± 0.1 mg cig^−1^ (ISO Intense smoking regime)^56^. Nicotine yields for different e-cigarette brands have been reported from 2 to 313 µg puff^−1^ while for conventional cigarettes smoke the values ranged from 170 to 232 µg puff^−1^^6,31,36,48,64^.

Nicotine-related impurities were present mainly in cigarette smoke at a significantly higher level than in e-cigarette emissions (note that the percentage reduction of anabasine and nicotine-*N*-oxide was not calculated because these impurities were < LOD in cigarette smoke and the e-cigarette emissions). In general, the nicotine used in e-liquids is extracted from tobacco and may contain other minor related alkaloids as impurities^64,65^. Therefore, nicotine-related impurities might be expected in e-cigarettes emissions and are considered acceptable by the USP and European Pharmacopeia in standard nicotine used in e-liquids^6,66–68^. The USP requires single impurities to be less than 0.5% (5 mg g^–1^) of nicotine, and total impurities to be less than 1% (10 mg g^–1^)^66^. The European Pharmacopeia requires each of seven specified impurities (anabasine, anatabine, cotinine, myosmine, nicotine-*N*-oxide, β-nicotyrine, nornicotine; Table 1) to be below 0.3%, unspecified impurities to be no more than 0.1% each, and total impurities to be less than 0.8% of nicotine content^6,66–68^. In our study, all analysed nicotine-related impurities in the e-cigarette emissions were below the levels stated by the USP and European Pharmacopeia for e-liquids (Table 1). This is consistent with the fact that only nicotine of pharmaceutical-grade is used in the production of these e-liquids. β-Nicotyrine, a pyrolysis product of nicotine^69^, was present at the highest level in cigarette smoke (0.42 µg puff^–1^). The observed reduction of 98% per puff in the emissions of both e-liquids is a good indication that the heat generated in the device atomiser is not sufficient to thermally breakdown nicotine to β-nicotyrine.

Another class of nicotine-related HPHCs of concern are tobacco-specific nitrosamines (TSNAs): namely, NNN, NNK, nitrosoanabasine and nitrosoanatabine. These non-volatile compounds may be present in e-liquids as impurities from tobacco nicotine extraction and are important compounds associated with negative health effects of cigarette smoke^34,70–74^. Two of the reported TSNAs, namely NNN and NNK, are classified as carcinogens and included in U.S FDA’s HPHC lists that apply to cigarette smoke and electronic nicotine delivery systems (ENDS)^39,53,75^. NNN and NNK are also included in the nine WHO TobReg priority smoke toxicants^55^. In our study, the levels of all four TSNAs were < LOD for both e-cigarette emissions with a percentage reduction of ≥ 99.9% as compared with cigarette smoke (Table 1).

### Polycyclic aromatic compounds

Another class of chemicals in cigarette smoke that poses health concerns are polycyclic aromatic hydrocarbons (PAHs), which are compounds with two or more fused benzenoid rings that are known for their carcinogenic and mutagenic properties^76^. The levels of PAHs in e-cigarette emissions were either < LOD or < LOQ (chrysene), except for naphthalene and pyrene (Table 2). Notably, these two compounds were higher than the limit of quantification (LOQ) in the method air blanks. Pyrene was in the same concentration in e-cigarettes as in the method air blank (0.1 ng puff^–1^). PAHs are present in the atmosphere as components of various dusts, tars, oils and engine exhaust gases^72^. The presence of pyrene in the e-cigarette aerosol is therefore most probably an artefact due to environmental contamination, as indicated by the method air blank.

**Table 2:** Polycyclic aromatic hydrocarbons: Per-puff concentration of e-cigarette vapour emissions, respective method air blanks and Ky1R6F cigarette smoke.

Vapour constituent	Unit	ePod1.0			Cigarette		Percentage reduction (%)
		Air blank	BB57	BB18	Ky1R6F	BB57	BB18
**Polycyclic aromatic hydrocarbons**							
Puff count	per consumable	50	50	50	9	-	-
Benzo[a]anthracene	ng puff^–1^	< LOD	< LOD	< LOD	3.38 ± 0.06	99.9	99.9
Benzo[a]pyrene	ng puff^–1^	< LOD	< LOD	< LOD	1.63 ± 0.04	99.7	99.7
Benzo[b]fluoranthene	ng puff^–1^	< LOD	< LOD	< LOD	1.64 ± 0.11	99.5	99.5
Benzo[c]phenanthrene	ng puff^–1^	< LOD	< LOD	< LOD	0.79 ± 0.02	99.7	99.7
Benzo[j]aceanthrylene	ng puff^–1^	< LOD	< LOD	< LOD	0.28 ± 0.01	96.9	98.2
Benzo[k]fluoranthene	ng puff^–1^	< LOD	< LOD	< LOD	0.51 ± 0.02	98.8	98.8
Chrysene	ng puff^–1^	< LOQ	< LOQ	< LOD	3.40 ± 0.06	99.8	99.9
Cyclopenta[c,d]pyrene	ng puff^–1^	< LOD	< LOD	< LOD	2.27 ± 0.11	99.8	99.8
Dibenzo[a,h]anthracene	ng puff^–1^	< LOD	< LOD	< LOD	0.15 ± 0.01	95.7	95.7
Dibenzo[a,e]pyrene	ng puff^–1^	< LOD	< LOD	< LOD	< LOQ	NC	NC
Dibenzo[a,h]pyrene	ng puff^–1^	< LOD	< LOD	< LOD	< LOQ	NC	NC
Dibenzo[a,i]pyrene	ng puff^–1^	< LOD	< LOD	< LOD	< LOQ	NC	NC
Dibenzo[a,l]pyrene	ng puff^–1^	< LOD	< LOD	< LOD	< LOQ	NC	NC
Indeno[1,2,3-cd]pyrene	ng puff^–1^	< LOD	< LOD	< LOD	0.68 ± 0.02	99.3	99.3
5-methylchrysene	ng puff^–1^	< LOD	< LOD	< LOD	0.02 ± 0.004	92.5	92.5
Naphthalene	ng puff^–1^	0.04 ± 0.01	0.18 ± 0.08	0.14 ± 0.10	131 ± 4.6	99.9	99.9
Pyrene	ng puff^–1^	0.10 ± 0.01	0.11 ± 0.01	0.10 ± 0.01	9.34 ± 0.12	98.9	98.9

Values are presented as mean ± standard deviation (n = 5). Percentage reduction of e-cigarette emissions relative to Ky1R6F cigarette smoke. *NC* not calculated, *LOD* limit of detection, *LOQ* limit of quantification. More information about the analytical methods and respective LODs and LOQs are under the Supplementary Information (Tables S1 and S2).

Levels of naphthalene were slightly higher than those of pyrene in e-cigarette emissions, while the respective air blank was lower. Nevertheless, it seems likely that these compounds were detected in e-cigarette emissions due to their presence as low-level contaminants in the background air, rather than originating from the vaping product. In terms of the levels detected, even if we assume a worst-case exposure of 300 puffs per day based on the million puff study (which reported a median use of 130 puffs day^–1^ and where 85% of users did not exceed 300 puffs day^–1^^77^), a consumer’s daily exposure to each of these compounds would be less than 0.15 µg day^–1^, the toxicological threshold of concern for mutagenic compounds^78,79^. Furthermore, most PAHs, including naphthalene and pyrene, showed a percentage reduction in e-cigarette emissions of ≥ 99% versus Ky1R6F cigarette smoke, while indenol[1,2,3-cd]pyrene showed a reduction of 92.5% because it was also present at only low levels in cigarette smoke (0.02 ng puff^–1^).

Collectively, our findings are consistent with the knowledge that PAHs are primarily products of combustion. For PAHs present at very high concentrations in cigarette smoke, such as benzo[a]pyrene, chrysene and pyrene, the percentage reduction in e-cigarette emissions was > 99%. In particular, benzo[a]pyrene, which is included in the nine WHO TobReg priority smoke toxicants, was reduced by 99.7% in e-cigarette emissions as compared with the smoke from the reference cigarette.

### Phenolic compounds and carbonyls

In cigarette smoke, the phenols of concern are catechol, *m*-cresol, *p*-cresol, *o*-cresol, hydroquinone, phenol and resorcinol (Table 3). They can be formed by the thermal degradation of tobacco leaf constituents such as lignin and chlorogenic acid^71,72,80,81^. Temperature is an important factor in the formation of phenolic compounds. Studies have reported that catechol and hydroquinone are formed in cigarette smoke at low temperatures (< 350 °C), while cresol, phenol and resorcinol are formed at temperatures of 350–600 °C^81^. In e-liquids, phenols and their precursors may be present as impurities derived from nicotine and may be transferred to the aerosol and inhaled by the vaper^71,72^. Phenols may also be formed upon vaporisation. Phenol emissions have been found to be independent of the nicotine benzoate concentration but significantly correlated with the PG/VG ratio. Emissions increased with power and puff duration, consistent with conditions that lead to a higher temperature and greater thermal degradation^82^. In our study, the levels of all seven phenols were < LOD in both e-cigarette emissions with a percentage reduction of ≥ 99% versus cigarette smoke (Table 3). The low operating temperatures of the e-cigarette device studied herein and the use of pharmaceutical- and food-grade ingredients in the e-liquids considerably reduce the likely presence of these phenolic compounds in e-cigarette aerosol.

**Table 3:** Phenolic compounds, carbonyls and ketones: per-puff concentration of e-cigarette vapour emissions, respective method air blanks and Ky1R6F cigarette smoke.

Vapour constituent	Unit	ePod1.0			Cigarette	Percentage reduction (%)	
		Air blank	BB57	BB18	Ky1R6F	BB57	BB18
**Phenolic compounds**							
Puff count	per consumable	50	50	50	9	-	-
Catechol	µg puff^–1^	< LOD	< LOD	< LOD	11.60 ± 0.62	> 99.9	> 99.9
*m*-Cresol	µg puff^–1^	< LOD	< LOD	< LOD	0.35 ± 0.02	99.8	99.8
*p*-Cresol	µg puff^–1^	< LOD	< LOD	< LOD	0.82 ± 0.05	99.9	99.9
*o*-Cresol	µg puff^–1^	< LOD	< LOD	< LOD	0.43 ± 0.04	99.8	99.8
Hydroquinone	µg puff^–1^	< LOD	< LOD	< LOD	11.40 ± 0.60	> 99.9	> 99.9
Phenol	µg puff^–1^	< LOD	< LOD	< LOD	1.67 ± 0.08	99.9	99.9
Resorcinol	µg puff^–1^	< LOD	< LOD	< LOD	0.39 ± 0.04	99.6	99.6
**Carbonyls (aldehydes and ketones)**							
Puff count	per consumable	50	50	50	9	-	-
Acetaldehyde	µg puff^–1^	< LOQ	0.10 ± 0.01	< LOQ	168 ± 17.2	99.9	> 99.9
Acetoin	µg puff^–1^	< LOD	< LOD	< LOD	1.25 ± 0.36	99.7	99.7
Acetone	µg puff^–1^	0.04 ± 0.003	0.04 ± 0.003	0.04 ± 0.002	65.8 ± 10.2	99.9	99.9
Acetyl propionyl	µg puff^–1^	< LOD	< LOD	< LOD	4.14 ± 0.67	> 99.9	> 99.9
Acrolein	µg puff^–1^	< LOD	< LOQ	< LOQ	18.2 ± 1.0	99.9	99.9
*n*-Butyraldehyde	µg puff^–1^	< LOD	< LOD	< LOD	4.26 ± 0.99	> 99.9	> 99.9
Diacetyl	µg puff^–1^	< LOD	< LOQ	< LOQ	27.8 ± 2.25	> 99.9	> 99.9
Crotonaldehyde	µg puff^–1^	< LOD	< LOD	< LOD	4.86 ± 0.70	99.9	99.9
Formaldehyde	µg puff^–1^	< LOQ	0.04 ± 0.004	0.07 ± 0.03	6.18 ± 0.68	99.4	98.8
Glyoxal	µg puff^–1^	0.01 ± 0.03	0.02 ± 0.002	0.05 ± 0.03	1.76 ± 0.11	99.1	97.0
Isobutyraldehyde	µg puff^–1^	< LOD	< LOD	< LOQ	6.50 ± 1.0	> 99.9	> 99.9
Methylglyoxal	µg puff^–1^	< LOQ	0.13 ± 0.02	0.19 ± 0.15	3.50 ± 1.20	96.3	94.5
Methyl ethyl ketone	µg puff^–1^	< LOD	< LOD	< LOD	19.5 ± 2.63	> 99.9	> 99.9
Propionaldehyde	µg puff^–1^	< LOQ	< LOQ	< LOQ	16.6 ± 2.1	99.9	99.9

Values are presented as mean ± standard deviation (n = 5). Percentage reduction of e-cigarette emissions relative to Ky1R6F cigarette smoke. *LOD* limit of detection, *LOQ* limit of quantification. More information about the analytical methods and respective LODs and LOQs are under the Supplementary Information (Tables S1 and S2).

Carbonyls in cigarette smoke are formed mainly by pyrolysis of tobacco sugars^83^, whereas those in e-cigarettes are formed mainly by thermal degradation of PG and/or VG^83–85^. Flavourings may also contribute to the formation of carbonyls, as well as the characteristics of the e-cigarette devices, especially the applied voltage, coil resistance and wicking material^47–49,86,87^. Poor wicking efficiency may lead to a dry wick and overheated e-liquid (dry puff), which promotes the formation of carbonyls and other toxic compounds^2,10,13,15^. Coil location, orientation, resistance and wick material, as well as power output, have been shown to affect carbonyl generation significantly^13,15,86^. E-liquid physical properties are also important in carbonyl formation^15,47,84,86^. The viscosity and density of the e-liquid determine its mobility, capillary action, and delivery to the wick and coil, influencing the likelihood of a dry puff^15^.

Several studies have reported the presence of carbonyls in e-cigarette emissions at levels ranging from 0.07 to 413 µg puff^–1^^85,88,89^. In our study, among the 14 analysed carbonyls (aldehydes and ketones), only five were quantifiable (acetaldehyde, acetone, formaldehyde, glyoxal and methylglyoxal) in the e-cigarette emissions at concentrations ranging from 0.02 to 0.19 µg puff^–1^ (Table 3). Of these, acetone was detected at the same level in e-cigarette emissions as the method air blank (0.04 µg puff^–1^). Detectable air blank values may arise from environmental contamination^6,9,34,90^. Acetaldehyde was quantified in BB57 emissions (0.10 µg puff^–1^) but was < LOQ in BB18 emissions, while formaldehyde was present in both (BB57, 0.04 µg puff^–1^; BB18, 0.07 µg puff^–1^). However, these two carbonyls were below the target levels proposed in the experimental voluntary standard published by the Association Française de Normalization (AFNOR; 16 µg puff^–1^ for acetaldehyde and 1 µg puff^–1^ for formaldehyde)^91^. Previous data indicate that the higher the percentage ratio of VG to PG, the higher the concentrations of carbonyl compounds emitted, especially acetaldehyde, acrolein and acetone^84^. In our study, both e-liquids had equivalent amounts of VG and PG; therefore, this ratio is likely to be irrelevant to the different concentrations of carbonyls detected in the two e-cigarette emissions, especially acetaldehyde. In a previous study, higher levels of acetaldehyde, acrolein and formaldehyde were generated in the emissions from an e-liquid without nicotine than in those from an e-liquid with nicotine; however, the observed carbonyl concentrations were strictly related to both the composition of the liquids and also the coil resistance^47^. In the presence of nicotine, the content of carbonyls, especially formaldehyde, was significantly higher with a 1.50-Ω coil than with a 0.25-Ω coil^47^. In our study, only acetaldehyde increased with the higher nicotine product (BB57); however, its concentration (0.10 µg puff^–1^) was still 160 times lower than the maximum level proposed by AFNOR (16 µg puff^–1^)^91^. A comparison of the emissions of an e-liquid with similar PG/VG ratio (1:1) emitted by a relatively similar Vype device (ePen) that uses a silica wick showed that formaldehyde at a concentration of 0.59 µg puff^–1^ was 8× higher and acetaldehyde at a concentration of 0.18 µg puff^–1^ was 2× higher than in the emissions presented herein (Table 3)^92^. In both studies, the values were below the maximum level proposed by AFNOR^91^.

From the studied carbonyls, only acetaldehyde, acrolein and formaldehyde are included in the nine WHO TobReg priority smoke toxicants^55^. Relative to cigarette smoke, their percentage of reduction in the e-cigarette emissions was ≥ 98.8%.

Among the 14 studied carbonyls, 7 were included in the new U.S. FDA HPHCs list for e-cigarettes^53,54^; namely, acetaldehyde, acrolein, formaldehyde and butyraldehyde, crotonaldehyde, and the diketones; diacetyl (2,3-butanedione) and acetyl propionyl (2,3-pentanedione). In the e-cigarette emissions, butyraldehyde and crotonaldehyde and acetyl propionyl were < LOD while acrolein and diacetyl were < LOQ. Acetoin, a precursor of diacetyl and acetyl propionyl, was also < LOD^93^.

Glyoxal and methylglyoxal are formed by thermal degradation or oxidation of PG and VG^87^. Glyoxal is considered mutagenic, while the related compound methylglyoxal has been identified as a metabolite during glycolysis and is thus naturally present in the body. Methylglyoxal is also present in foods and drinks such as honey and coffee. A lack of data has led to classification of methylgloxal as a Group 3 carcinogen (carcinogenicity to humans not classifiable) by IARC. Both compounds have been previously detected in e-cigarette emissions at concentrations of 0.07–0.94 and 0.09–33 µg puff^–1^, respectively^86,88^. In our study, glyoxal was present at lower levels (BB57 and BB18, 0.02 and 0.05 µg puff^–1^, respectively), while methylglyoxal was at concentrations of 0.13 and 0.19 µg puff^–1^ in BB57 and BB18, respectively (Table 3). Glyoxal was detected in the method air blank and therefore the actual levels in the e-cigarette emissions are potentially lower than reported in Table 3. Again assuming a worst-case exposure of 300 puffs day^–1^ spread over 8 h, the levels of glyoxal exposure to a consumer would still be more than 40 times lower than the occupational exposure limit of 0.10 mg m^–3^^77,94,95^. The high standard deviation for glyoxal and methylglyoxal observed in BB18 e-cigarette emissions is probably related to an analytical sample matrix effect and/or batch variability^83,96,97^. Despite the high standard deviation, the percentage reduction of glyoxal and methylglyoxal in both e-cigarette emissions relative to cigarette smoke was ≥ 97.0% and ≥ 94.5%, respectively (Table 3).

In our analysis, levels of carbonyls were considerably reduced relative both to other studies of e-cigarettes and to Ky1R6F cigarette smoke. Levels below the LOD or LOQ, or even below the threshold levels proposed by the AFNOR standard guidelines, provide evidence of the optimal operation conditions (e.g., adequate wick saturation without extreme coil heating) of the ceramic wick-based device.

### Volatile organic compounds

Table 4 summarizes the levels of volatile organic compounds (VOCs) in the e-cigarette emissions and Ky1R6F cigarette smoke, along with the percentage reductions. Among the 23 VOCs analysed, levels were < LOD for both e-cigarette emissions, except for hydrogen cyanide (BB57, < LOQ), allyl alcohol (both < LOQ) and acetamide (BB57, < LOQ). Four of the VOCs, namely, acrylonitrile, benzene, propylene oxide and toluene, are listed by the U.S. FDA as compounds of concern for e-cigarettes^53^, while benzene and 1,3-butadiene are included in the nine WHO TobReg priority smoke toxicants^55^. The level of all of these compounds was < LOD with percentage reductions of ≥ 99.0% relative to Ky1R6F smoke (Table 4).

**Table 4:** Volatiles compounds: Per-puff concentration of e-cigarette vapour emissions, respective method air blanks and Ky1R6F cigarette smoke.

Vapour constituent	Unit	ePod1.0			Cigarette	Percentage reduction (%)	
		Air blank	BB57	BB18	Ky1R6F	BB57	BB18
**Volatile organic compounds**							
Puff count	Per consumable	50	50	50	9	-	-
Acetamide	µg puff^–1^	< LOD	< LOQ	< LOD	1.46 ± 0.09	99.4	99.8
Acrylamide	µg puff^–1^	< LOD	< LOD	< LOD	0.48 ± 0.03	98.7	98.7
Acrylonitrile	µg puff^–1^	< LOD	< LOD	< LOD	2.24 ± 0.24	99.9	99.9
Allyl alcohol	µg puff^–1^	< LOD	< LOQ	< LOQ	1.38 ± 0.18	99.7	99.8
Benzene	µg puff^–1^	< LOD	< LOD	< LOD	8.46 ± 0.85	> 99.9	> 99.9
Benzo(b)furan	µg puff^–1^	< LOD	< LOD	< LOD	0.07 ± 0.01	99.3	99.3
1,3-Butadiene	µg puff^–1^	< LOD	< LOD	< LOD	9.35 ± 0.60	> 99.9	> 99.9
Ethylbenzene	µg puff^–1^	< LOD	< LOD	< LOD	1.40 ± 0.23	99.9	99.9
Ethyl carbamate	ng puff^–1^	< LOD	< LOD	< LOD	< LOD	NC	NC
Ethylene oxide	µg puff^–1^	< LOD	< LOD	< LOD	1.84 ± 0.13	99.8	99.8
Hydrogen cyanide	µg puff^–1^	< LOD	< LOQ	< LOD	42.5 ± 1.86	99.9	99.9
Hydrazine	ng puff^–1^	< LOD	< LOD	< LOD	< LOD	NC	NC
Furan	µg puff^–1^	< LOD	< LOD	< LOD	6.09 ± 0.47	> 99.9	> 99.9
Isoprene	µg puff^–1^	< LOD	< LOD	< LOD	90.6 ± 7.18	> 99.9	> 99.9
Nitrobenzene	µg puff^–1^	< LOD	< LOD	< LOD	< LOD	NC	NC
Nitromethane	ng puff^–1^	< LOD	< LOD	< LOD	46.2 ± 5.6	98.2	98.2
Propylene oxide	ng puff^–1^	< LOD	< LOD	< LOD	156 ± 26.4	99.0	99.0
Pyridine	µg puff^–1^	< LOD	< LOD	< LOD	2.19 ± 0.25	99.9	99.9
Quinoline	µg puff^–1^	< LOD	< LOD	< LOD	0.06 ± 0.003	99.4	99.4
Styrene	µg puff^–1^	< LOD	< LOD	< LOD	1.10 ± 0.08	99.9	99.9
Toluene	µg puff^–1^	< LOD	< LOD	< LOD	13.3 ± 1.65	> 99.9	> 99.9
Vinyl acetate	ng puff^–1^	< LOD	< LOD	< LOD	58.6 ± 2.39	98.1	98.1
Vinyl chloride	ng puff^–1^	< LOD	< LOD	< LOD	11.2 ± 0.88	99.4	99.4

Values are presented as mean ± standard deviation (n = 5). Percentage reduction of e-cigarette emissions relative to Ky1R6F cigarette smoke. *NC* not calculated, *LOD* limit of detection, *LOQ* limit of quantification. More information about the analytical methods and respective LODs and LOQs are under the Supplementary Information (Tables S1 and S2).

In particular, benzene, which may be formed by decarboxylation of benzoic acid, was undetectable in both e-cigarette emissions. Pankow et al*.*^98^ previously reported that benzene concentrations were largely undetectable for an e-cigarette with a single vertical coil and a cotton wick, but were more readily detected for a device with a single horizontal coil and a silica wick. Their results demonstrated the importance of the orientation of the coil and the type of wicking material in the formation of benzene. Our results showed that neither use of benzoic acid in the BB18 formulation nor the characteristics or operating conditions of the device contributed to benzene formation in the e-cigarette emissions. Pankow et al*.*^98^ also reported that benzene may be formed by the dehydration and cyclization of PG and VG, especially at high-power settings using a tank system with a single horizontal coil and a silica wick. Other studies have shown that 1,3-butadiene may be formed by VG degradation and is an important intermediate in the formation of benzene from VG^84,98^. Aromatic VOCs such as toluene, xylene, styrene and ethylbenzene may also be formed by thermal degradation of VG, where benzene plays an important role as an intermediate^84^. Benzene and other combustion-related compounds including acrylonitrile, isoprene and toluene may also be present in e-cigarette emissions as impurities of nicotine^34^. Percentage transfers to aerosol of ≥ 89% have been reported for these compounds after fortification of e-liquids at high levels (46–232 ng g^–1^)^34^. Other combustion-related compounds such as allyl alcohol and propylene oxide have been detected in e-cigarette emissions as thermal degradation products of PG and/or VG^49,99^. In our study, all these compounds were < LOD or < LOQ.

The above-cited studies show that the chemical composition of the e-liquid, the design of the device, and the temperature at which the e-liquids vaporize have a strong impact on the formation of VOCs and their transfer to e-cigarette emissions, especially those that originate primarily from heating of PG and VG. Our findings of levels < LOD or < LOQ in the e-cigarette emissions for the studied VOCs indicate the consistent supply of e-liquid by the ceramic wick without overheating of the coil and, consequently, overheating of the e-liquid. There was no considerable difference in levels of VOCs in the two emissions produced from e-liquids with different types of nicotine salt and different nicotine strengths. Moreover, there was a considerable reduction of VOCs in both e-cigarette emissions relative to cigarette smoke.

### Flavouring compounds and acids

Next, we examined the e-cigarette emissions of flavouring compounds, together with acetic acid and propionic acid, as listed by the U.S. FDA as HPHCs of concern for e-cigarettes^53,54^. These compounds were not analysed in cigarette smoke because the Ky1R6F cigarette used in the study is an unflavoured US-blended cigarette and validated analytical methods for these compounds were not available.

All compounds were < LOD or < LOQ except for isoamyl acetate (isopentyl acetate) in the e-cigarette emissions (Table 5). This flavouring compound was used in both e-liquid formulations (BB57 and BB18), and therefore its presence in the e-cigarette emissions was expected. In a quantitative risk estimation performed in line with a published approach to the risk assessment of flavours in e-liquids^100^, the level of isoamyl acetate in the formulation was found to be supportable even if 100% of it were transferred to the aerosol. To further establish the level of risk, we again assumed a worst-case exposure of 300 puffs day^–1^ over 8 h^77^, which would result in isoamyl acetate exposure levels of 0.23 mg day^–1^ or 0.034 mg m^–3^. This is several orders of magnitude below various occupational exposure guidelines for isoamyl acetate, the lowest of which is 250 mg m^–3^, and below the acceptable daily intake of 3 mg kg^–1^ day^–1^ (180 mg day^–1^ for a 60-kg adult) established by the Joint FAO/WHO Expert Committee on Food Additives^101,102^.

**Table 5:** Flavouring compounds and acids: Per-puff concentration of e-cigarette vapour emissions and respective method air blanks.

Vapour constituent	Unit	ePod1.0		
		Air blank	BB57	BB18
**Flavouring compounds**				
Puff count	Per consumable	50	50	50
1-Butanol	µg puff^–1^	< LOD	< LOQ	< LOQ
Benzyl acetate	µg puff^–1^	< LOD	< LOD	< LOD
Ethyl acetate	µg puff^–1^	< LOD	< LOD	< LOD
Ethyl acetoacetate	µg puff^–1^	< LOD	< LOD	< LOD
Furfural	µg puff^–1^	< LOD	< LOD	< LOD
Isoamyl acetate	µg puff^–1^	< LOD	0.76 ± 0.16	0.62 ± 0.04
Isobutyl acetate	µg puff^–1^	< LOD	< LOD	< LOD
Methyl acetate	µg puff^–1^	< LOD	< LOD	< LOD
**Acids**				
Puff count	Per consumable	50	50	50
Acetic acid	µg puff^–1^	< LOD	< LOD	< LOD
Propionic acid	µg puff^–1^	< LOD	< LOQ	< LOD

Values are presented as mean ± standard deviation (n = 5). *LOD* limit of detection, *LOQ* limit of quantification. More information about the analytical methods and respective LODs and LOQs are under the Supplementary Information (Tables S1 and S2).

### Study limitations

The aim of this study was to undertake an assessment of the emissions generated by an e-cigarette using a ceramic wick-based technology and the comparison with conventional cigarette smoke. The generation of emissions from the e-cigarettes followed ISO 20768:2018 (55 mL puff volume, 3 s puff duration, 30 s puff frequency)^52^. ISO 20768:2018 lays out the essential requirements/conditions necessary to generate and collect e-cigarette emissions for analytical and comparison purposes in a robust and reproducible manner. The standard was developed building on the CORESTA (Cooperation Centre for Scientific Research Relative to Tobacco) recommended method no. 81 for machine puffing of e-cigarettes^45,103^. It is recognised that no single puffing regime can reflect the wide range of consumers’ puffing behaviour expected with e-cigarette use however, the use of ISO 20768:2018 is important for cross-product comparative purposes^45^. The application of different types of regime and its impact on the device performance is out of scope of this study. Nevertheless, the applied ISO standard puffing regime demonstrated that the emissions collected as block of 50 sequential puffs contained low levels of carbonyl compounds which would be associated with the thermal degradation of PG and VG. The carbonyl levels from the studied e-cigarettes emissions were either below the LOD or LOQ, or below the threshold levels proposed by the AFNOR standard guidelines, which provides evidence of the adequate wicking rate of the ceramic block without extreme coil heating under the test conditions. Whilst data generated from the first 50 puffs may not represent yields over the range of all puffs, especially as the liquid becomes depleted, the data are representative to enable generalised comparisons.

## Conclusions

To follow a strategy of tobacco harm reduction, it is important to continually characterise the vapour emitted by newly developed e-cigarette devices relative to the smoke from combusted cigarettes in order to understand the chemical composition of the emissions. In this study, the focus was on the quantification of a wide range of HPHCs in the combustible cigarette smoke and the e-cigarette vapour emissions from the new pod/cartridge generation e-cigarettes using a ceramic-wick based technology. The higher nicotine emissions for BB57 compared with BB18 were not associated with a notable increase in the amounts of any of the quantified HPHCs. A substantial reduction of the levels of studied HPHCs and the nine TobReg priority smoke toxicants in the e-cigarette emissions relative to Ky1R6F combustible cigarette smoke was observed, with percentage of reductions in the range from 81.6% to > 99.9%. The low or undetectable levels of these compounds in e-cigarette emissions may be attributed to (1) the low operating temperature (< 350 °C) of the device; (2) an efficient supply of e-liquid by the ceramic wick to the heating coil without overheating of the coil or e-liquid; and (3) the use of pharmaceutical- or food-grade e-liquid ingredients. While the e-cigarette tested is unlikely to be risk-free, the results demonstrate that this ceramic wick-based device can offer considerably lower toxicant exposure when compared with combustible cigarettes under the tested conditions used in the study. Further pre-clinical in vitro, clinical and population studies are needed to evaluate the exposure of those toxicants and associated risks at an individual and populational level.

## Methods

### E-cigarette device

**Figure 1:**
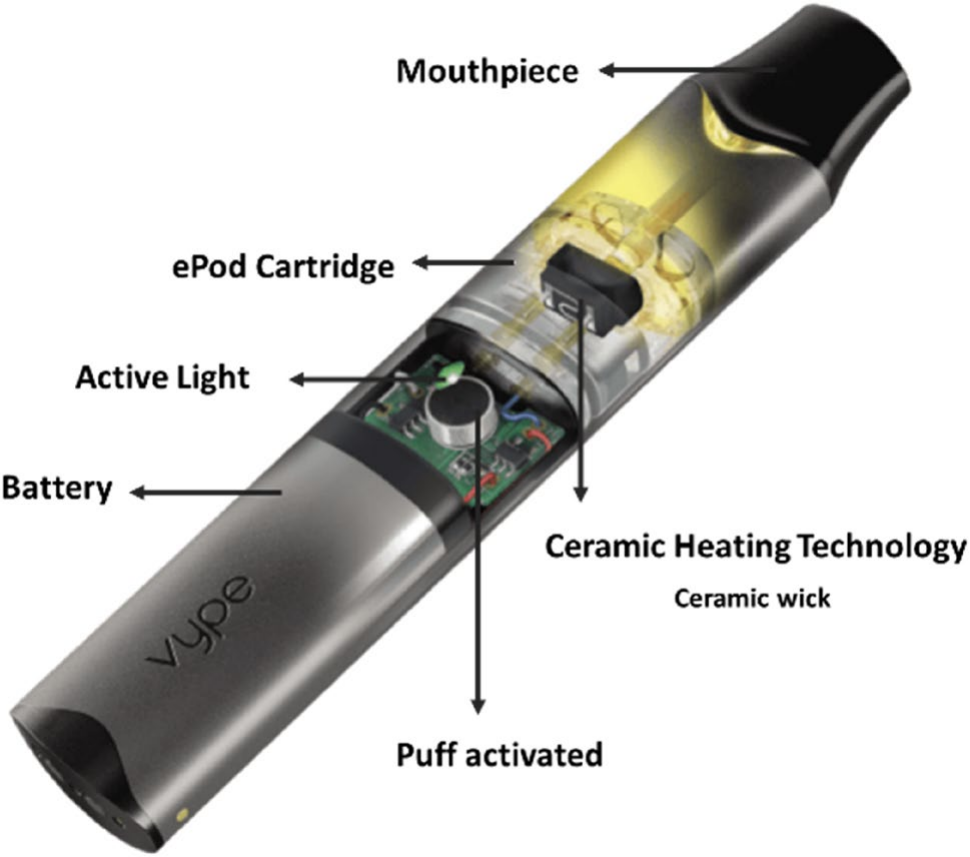
Main components of the Vype e-cigarette device.

The e-cigarette device (Vype ePod1.0, Nicoventures Trading Ltd., Blackburn, UK) consists of a metallic outer device case, a printed circuit board to control the device, a lithium-ion rechargeable battery (350 mAh) and an e-cigarette cartridge (Fig. 1). The voltage ranges from 2.2 to 3.1 V and is not adjustable by the user. The device has dimensions (h × w × d) of 104.2 × 19.1 × 10.5 mm and a power output of 6.5 ± 0.5 W. The electronic parts are switched on when a puff is taken. The cartridges or pods consist of a plastic case holding the ceramic wick material and a flat metal heating element (NiCr, 0.8 –1.4-Ω resistance). Each pod is pre-filled with Vype e-liquid (1.9 mL) and is magnetically attached to the device.

### E-liquids

The two e-liquids tested in the study were Berry Blast flavour with nicotine levels of 57 and 18 mg mL^–1^. Both e-liquids contained equivalent amounts of VG and PG (50:50, %w/w). Berry Blast 57 mg mL^–1^ (BB57) contained lactic acid, while Berry Blast 18 mg mL^–1^ (BB18) contained benzoic acid.

### Ky1R6F reference standard cigarettes

The tobacco cigarette used as a comparator was the Kentucky Reference Cigarette 1R6F (Centre for Tobacco Reference Products, University of Kentucky, USA), which has been designed to provide a standard test piece for scientific studies. It is an unflavoured US-blended king-sized product with a cellulose acetate filter, an aerosol nicotine level of 1.9 ± 0.1 mg cig^–1^, and a tar yield of 29 ± 2 mg cig^−1^ as measured by the ISO Intense smoking regime^6,56^. At present, reference products for e-cigarette testing are not available.

### Sample generation—smoking and puffing conditions

Sample generation and emissions testing were conducted by Labstat International ULC (Labstat, Kitchener, Ontario, Canada). Cigarettes were conditioned at a temperature of 22 ± 2 °C and a relative humidity of 60 ± 3% for at least 48 h as per ISO 3402^104^. Prior to testing, the reference Ky1R6F cigarettes were marked with the standard butt length specified by ISO 4387^105^. Smoking and puffing parameters and smoking machine specifications are summarized in Table 6.

**Table 6:** Emissions testing parameters for the reference cigarette and the e-cigarette e-liquids.

Study product	Puffing regime^a^	Device angle	Puff number	Replicates	Refs
Ky1R6F cigarette	55/2/30; bell-shaped puff profile, 100% ventilation block (ISO 20778:2018)^57^	N/A	To butt mark (9 on average)	5	^56,57,104–106^
**Vype ePod**					
Berry Blast, 57 mg mL^–1^	55/3/30; rectangular- puff profile (ISO 20768:2018)^52^	15° battery side down	50	5	^52^
Berry Blast, 18 mg mL^–1^	55/3/30; rectangular puff profile (ISO 20768:2018)^52^	15° battery side down	50	5	^52^

^a^Puffing regime: volume (mL)/puff duration (s)/puff frequency (s).

Cigarettes were smoked under the ISO intense smoking regime to the butt mark with filter ventilation blocked (typically 9–10 puffs)^40,57,106^. E-cigarettes were puffed according to ISO 20768:2018^52^. Smoking of cigarettes and puffing of e-cigarettes were carried out in dedicated conditioned rooms^104^ using either a rotary or a linear smoking machine^52,57,106^. Cigarette smoke and e-cigarette emissions were sampled/analysed as five independent replicates.

### Analytical methods

The analytical methods used by Labstat International ULC (Labstat, Kitchener, Ontario, Canada) are described in Supplementary Information, Table S1. In total, 23 different analytical methods were used to quantify 89 target analytes in the emissions from e-cigarettes and/or in mainstream Ky1R6F cigarette smoke. The methods used were largely based on Health Canada methods for cigarette smoke analysis, with additional methods developed by Labstat for other HPHCs and e-cigarette compounds of interest^6^. The methods were adapted for use with e-cigarettes where necessary. The operation of the methods is accredited to ISO/IEC 17025:2017^107^ for all reported constituents of mainstream tobacco smoke and e-cigarette aerosols, except where noted in Supplementary Table S1. Air (method) blank determinations were also conducted for e-cigarette emissions in order to identify background contaminants or other interference. The method LODs and LOQs are summarised in the Supplementary Information, Table S2.

### Data analysis—percentage reduction

The percentage reduction in e-cigarette emissions was calculated relative to the Ky1R6F reference cigarette. For this calculation, the average of 5 replicate measurements for each product was used. For some toxicants, the level in the e-cigarette emissions was < LOD and/or < LOQ. In cases where the emissions were < LOD, the imputed value was LOD/2^6,107^. For data < LOQ but > LOD, the imputed value was calculated as the midpoint between the reported LOD and LOQ^6,108^. Imputation was carried out on an individual replicate bases prior to calculating averages. In cases where both the e-cigarette and the reference combustible cigarette (Ky1R6F) emission levels were < LOQ or < LOD, the percentage reduction was not calculated (NC). LOD and LOQ for each compound for the e-cigarette emissions and cigarette smoke are reported in Supplementary Table S2.

## Supplementary Information

Supplementary Tables.

## Data Availability

Data are available from the authors on request. Any inquiries can be directed to the corresponding author.
